# Emergence of Recombinant SARS-CoV-2 Variants in California from 2020 to 2022

**DOI:** 10.3390/v16081209

**Published:** 2024-07-27

**Authors:** Rahil Ryder, Emily Smith, Deva Borthwick, Jesse Elder, Mayuri Panditrao, Christina Morales, Debra A. Wadford

**Affiliations:** 1 Viral and Rickettsial Disease Laboratory, California Department of Public Health, Richmond, CA 94804, USA; 2 Theiagen Genomics, Highlands Ranch, CO 80129, USA; emily.smith@theiagen.com; 3 COVID Control Branch, Division of Communicable Disease Control, CDPH, Richmond, CA 94804, USA

**Keywords:** SARS-CoV-2, genomic epidemiology, genomic surveillance, recombination, whole-genome sequencing, California COVIDNet

## Abstract

The detection, characterization, and monitoring of SARS-CoV-2 recombinant variants constitute a challenge for public health authorities worldwide. Recombinant variants, composed of two or more SARS-CoV-2 lineages, often have unknown impacts on transmission, immune escape, and virulence in the early stages of emergence. We examined 4213 SARS-CoV-2 recombinant SARS-CoV-2 genomes collected between 2020 and 2022 in California to describe regional and statewide trends in prevalence. Many of these recombinant genomes, such as those belonging to the XZ lineage or novel recombinant lineages, likely originated within the state of California. We discuss the challenges and limitations surrounding Pango lineage assignments, the use of publicly available sequence data, and adequate sample sizes for epidemiologic analyses. Although these challenges will continue as SARS-CoV-2 sequencing volumes decrease globally, this study enhances our understanding of SARS-CoV-2 recombinant genomes to date while providing a foundation for future insights into emerging recombinant lineages.

## 1. Introduction

SARS-CoV-2 recombinants present unique challenges for public health genomic surveillance systems regarding detection, characterization, and possible impacts on the phenotype of the virus. Recombinant SARS-CoV-2 genomes arise through the recombination of at least two different SARS-CoV-2 lineages and generally emerge following the co-circulation of multiple lineages at high prevalence. Recombination events likely occur within a single patient co-infected with co-circulating lineages [1,2,3]. However, there is also genomic evidence suggesting that recombination between non-co-circulating viruses may occur within long-term-infected individuals, wherein the original lineage recombines with a more recent lineage from a subsequent infection [4]. 

Recombinant genomes are important to track because they contain a novel combination of mutations not seen previously in a single lineage, such as the XBC recombinant, which includes mutations from both the Delta and Omicron (BA.2) variants (https://outbreak.info/compare-lineages, accessed on 4 March 2024). Recombination between SARS-CoV-2 genomes can result in the creation of novel strains with the potential to outcompete other lineages and evade host immunity, which has implications for both vaccine development for and the treatment of SARS-CoV-2 [5,6]. It is also thought that recombination between different coronaviruses played a role in the origin of SARS-CoV-2, further demonstrating the importance of monitoring recombinant viruses in a public health context [7,8,9]. Prior studies have found higher rates of hospitalization and lower neutralization titers for certain SARS-CoV-2 recombinant genomes [10,11]. However, the lack of available epidemiological metadata in public sequence repositories makes a large-scale analysis of these associations a significant challenge.

Worldwide, the first recognized SARS-CoV-2 recombinant lineage, designated as XA, was formed between lineages B.1.1.7 and B.1.177 and emerged in Europe in early 2021 following the co-circulation of those lineages in that geographic region (https://virological.org/t/recombinant-sars-cov-2-genomes-involving-lineage-b-1-1-7-in-the-uk/658, accessed on 4 March 2024). However, a previous study revealed that SARS-CoV-2 recombinants were likely circulating at low levels early in the pandemic prior to the very first recombinant Pango lineage designation [12]. Through the end of 2022, 60 recombinant lineages were designated, some of which disseminated worldwide, while others remained as local clusters. 

Throughout the pandemic, many designated recombinant SARS-CoV-2 lineages and several novel recombinant lineages were identified using California genomic surveillance data. The high sequencing volume of SARS-CoV-2-positive specimens in California allowed for the detection of emerging lineages not yet at high prevalence [13].

The objective of this study was to provide an overview of the genomic landscape of SARS-CoV-2 recombinants in California from the beginning of the pandemic through 31 December 2022 in combination with epidemiologic metadata to gain insight into how SARS-CoV-2 recombinant lineages emerge and spread within a population. Additionally, this study highlights persistent challenges in monitoring the emergence of novel SARS-CoV-2 lineages via genomic surveillance.

## 2. Materials and Methods

### 2.1. Genome Inclusion Criteria

The recombinant genomes examined in this study were obtained from the California COVIDNet sequence database [13] hosted on Terra, a cloud-based bioinformatics platform used for genomic surveillance across many different public health laboratories in California [14], and GISAID [15,16,17]. A genome was included in this study if it was collected in California prior to 1 January 2023 and determined to be a recombinant of 2 or more SARS-CoV-2 lineages. Genomes were determined to be recombinant either manually or systematically. The manual process employed involved identifying groups of mutations within the same genome that defined two or more clades according to CoVariants (https://covariants.org/variants, accessed 4 March 2024), following guidance developed by the Public Health Alliance for Genomic Epidemiology (https://github.com/pha4ge/pipeline-resources/blob/main/docs/sc2-recombinants.md, accessed 4 March 2024). This manual process was performed for all putative novel recombinants. The systematic process for identifying recombinant lineages was simply the detection of genomes with a resulting Pango lineage beginning with an “X”, as per the Pango Lineage Nomenclature (https://virological.org/t/pango-lineage-nomenclature-provisional-rules-for-naming-recombinant-lineages/657, accessed 4 March 2024) [18,19]. Genomes from the California COVIDNet sequence database were included if they had greater than 85% breadth of coverage relative to Wuhan-1 (NC_045512) as determined by the TheiaCoV workflows within the Public Health Bioinformatics Github repository (https://github.com/theiagen/public_health_bioinformatics, accessed 4 March 2024). Duplicate genomes in both the California COVIDNet sequence database and GISAID were removed by matching internal identifiers to the Virus Name in GISAID. Some genomes were in the California COVIDNet sequence database but not GISAID due to either the location information being considered identifiable or because GISAID had rejected the submitted genomes.

### 2.2. Genomic Epidemiology 

To match SARS-CoV-2 sequences to epidemiologic information, data were obtained from four different California Department of Public Health (CDPH) databases: the COVID-19 Hospitalization Registry, the COVID-19 Case Registry, the Vaccine Registry, and the Integrated Genomic Epidemiology Dataset (IGED). The COVID-19 Hospitalization Registry includes data reported to CDPH following an All Facilities Letter (AFL) that required all hospitals in California to report specific patient-level information for each hospitalized patient who tested positive for COVID-19 on 27 July 2021 or later. The COVID-19 Case Registry includes SARS-CoV-2 laboratory results that have been reported electronically or manually by laboratories, healthcare providers, and local health departments. The Vaccine Registry database includes vaccine information reported to the California Immunization Registry (CAIR). The IGED is a database of California SARS-CoV-2 lineages derived from whole-genome sequencing (WGS) along with case demographic and epidemiologic information from the COVID-19 Case Registry. SARS-CoV-2 sequence data are required to be reported to CDPH as per the updated California Code of Regulations Title 17, Section 2505, subsection (q). 

A case was considered vaccinated if a dose of COVID-19 vaccine was administered 14 days or more before the earliest known date associated with a specimen. An unknown vaccination status and unvaccinated status were not distinguishable and therefore not included. The denominator used to calculate percentages of cases that matched with the aforementioned CDPH databases was the total count of recombinant genomes, while the denominator used to determine percentage of hospitalizations and deaths was the total number of matched cases.

To compare total California sequences of all SARS-CoV-2 lineages in the same study period, analyses were performed for all California de-duplicated sequences using the above-mentioned databases via the following variables of interest: Specimen Collection Month, Vaccination Dose Count, Deaths, and Hospitalization. Specimen Collection Month was derived from the earliest date associated with each specimen and taken from the WGS dataset along with Pango Lineage and WHO variant classification. Total case counts were taken from the California COVID-19 Case Registry and included only confirmed cases. A confirmed case was defined as an individual in whom SARS-CoV-2 ribonucleic acid (RNA) was detected in a clinical or post-mortem specimen using a diagnostic molecular amplification test performed by a Clinical Laboratory Improvement Amendments (CLIA)-certified provider or the detection of SARS-CoV-2 RNA in a clinical or post-mortem specimen via genomic sequencing. A map of the number of recombinants per region with population size (worldpopulationreview.com accessed 4 March 2024) was created using ArcGIS Pro (version 3.0.0).

### 2.3. Phylogeny and Recombinant Sites

Phylogenetic trees of select recombinant lineages, including both California and non-California genome assemblies, were obtained by uploading GISAID accessions to UShER for phylogenetic placement [20]. Separate analyses were performed specifically on California genomes using the Augur workflows (https://github.com/theiagen/public_health_bioinformatics, accessed 4 March 2024) with default settings on Terra.bio, which uses the StaPH-B docker image (https://github.com/StaPH-B/docker-builds/tree/master/augur, accessed 4 March 2024) of the Nextstrain Augur tool (https://docs.nextstrain.org/projects/augur/en/stable/index.html accessed 4 March 2024) for phylogenetic tree construction [21]. Auspice was used for tree visualization to demonstrate spread throughout the state of California [19]. Genomes were annotated according to the corresponding California Public Health Officer Region if location information was available (https://www.cdph.ca.gov/Programs/CID/DCDC/Pages/COVID-19/Order-of-the-State-Public-Health-Officer-Hospital-Health-Care-System-Surge-FAQ.aspx, accessed 4 March 2024).

Recombinant sites within the genome were identified based on the original Github issue on the pango-designation repository (https://github.com/cov-lineages/pango-designation/, accessed 4 March 2024) for designated recombinant lineages or determined empirically by manually assessing the mutation pattern for novel recombinant lineages. Microsoft PowerPoint was used to create a figure approximating the recombinant sites across the SARS-CoV-2 genome for the recombinant lineages included in this study.

## 3. Results

### 3.1. Landscape of SARS-CoV-2 Recombinants in California

Through December 31, 2022, 4213 SARS-CoV-2 recombinant genome sequences that originated from specimens collected in California were identified (Supplementary Table S1). Of these, 2889 (68.6%) were hosted on Terra in the California COVIDNet sequence database, and 1324 (31.4%) were identified based on GISAID. Among the recombinants from the California COVIDNet sequence database and GISAID, 3932 (93.3%) belonged to a designated SARS-CoV-2 recombinant lineage, while the remainder belonged to novel recombinant lineages. The major identified recombinant lineages (>30 sequences) were primarily composed of Omicron parental lineages, except for XB (B.1.634 x B.1.631), a recombinant of two lineages that did not belong to one of the WHO variant classifications. 

**Figure 1:**
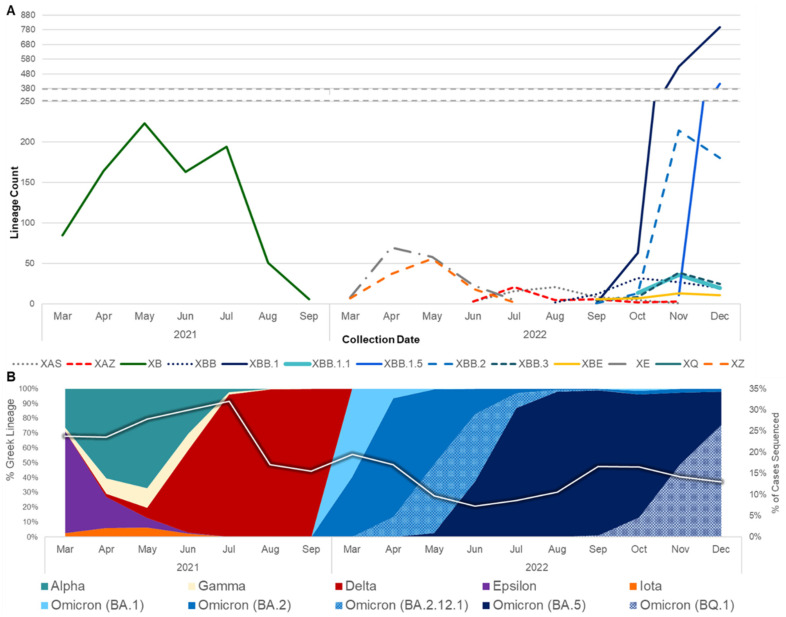
Prevalence of SARS-CoV-2 recombinants relative to SARS-CoV-2 variants from March 2021 to December 2022. (**A**) The number of genomes belonging to major recombinant lineages (*n* ≥ 30) in California. (**B**) The prevalence of WHO variants and Pango lineage groupings in California data available from the California COVIDNet sequence database in Terra (left *y*-axis). The white line represents the percentage of COVID-19 cases sequenced in California over time (right *y*-axis).

The first recombinant lineage detected in California was XB, collected on 3 May 2021, when the prevalent lineages were primarily Alpha (B.1.1.7) and Epsilon (B.1.427/B.1.429) variants (Figure 1). XB circulated in California through September 2021, at which time Delta had become the dominant variant in this state. Interestingly, none of the designated Delta × Omicron (BA.1) recombinant lineages, XD or XF, were identified in California, but two novel Delta × BA.1 recombinant genomes were identified between December 2021 and February 2022. In the following months, BA.1 was rapidly replaced by BA.2, leading to the designation of more than 30 recombinant BA.1 × BA.2 lineages, most of which were ultimately detected in California. XE was the most frequently identified of these BA.1 × BA.2 recombinants, followed by XZ. At least three novel recombinant BA.1 × BA.2 lineages were also detected in California, but none of those lineages grew beyond 50 genomes, so they did not receive a Pango lineage designation. Not long after BA.2 became the dominant lineage in California, BA.5 emerged, giving rise to a new set of recombinants with BA.2 and BA.5 parental lineages. XAS is the most frequently identified BA.5 × BA.2 recombinant lineage to date in California.

The rise of second-generation BA.2 lineages such as BA.2.75, BA.2.3.20, and BA.2.10.1, which trailed the initial BA.2 surge earlier in 2022 by several months, gave rise to another set of BA.2 and BA.5 recombinants. This set included XBD, XBF, XBJ, and others, all of which were identified in California and remained in circulation through the end of 2022. Similarly, two second-generation BA.2 lineages, BA.2.75 and BA.2.10.1, recombined to form the XBB lineage, of which 2044 (constituting 48.5% of the recombinants surveyed) were identified in California. Diversification of the XBB recombinant gave rise to XBB.1.5, a sublineage that was projected to become dominant in the United States shortly after emergence, of which 419 (amounting to 9.9% of the recombinants surveyed) were identified in California through the end of 2022. Two Delta × BA.2 recombinant lineages, XAY and XBC, were identified in California beginning in September 2022 and remained in circulation through December 2022. 

**Figure 2:**
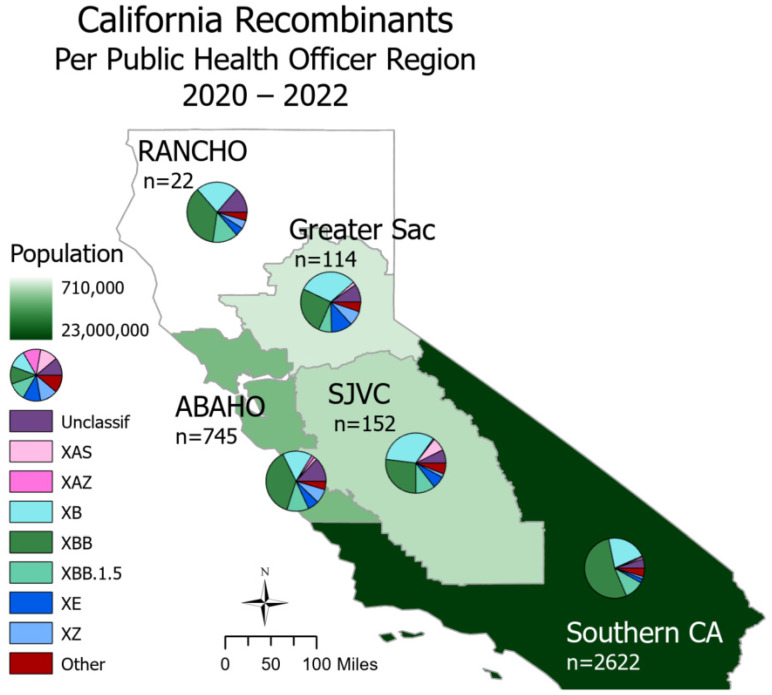
Recombinant lineages in California from 2020 to 2022 classified by Public Health Officer Region (PHO). Total sequence count (*n*) is split via a pie chart according to major recombinant lineages, and the “Other” category includes lineages with counts less than 30. PHO regions are colored according to 2023 population size. There are 558 sequences with unknown county information; these are not depicted on this map.

SARS-CoV-2 recombinants were spread throughout all regions of California (Figure 2). In Northern California, the Rural Association of Northern California Health Officers (RANCHO) and the Association of Bay Area Health Officials (ABAHO) reported that XBB accounted for the largest proportion of recombinants (36.4–37.9%). For the inland regions, represented by Greater Sacramento and the San Joaquin Valley Consortium (SJVC), the majority of recombinants were XB (31.6–32.9%) and XBB (25.4–27.0%). In the Southern California region, XBB accounted for 53.2% of all recombinants. 

### 3.2. Epidemiological Data from CDPH Databases

Among the California SARS-CoV-2 recombinant genomes, 2523 (59.9%) matched to CDPH COVID-19 Hospitalization Registry and COVID-19 Case Registry databases (Table 1). Of these matched cases, 228 (5.4%) were less than 17 years old, 1524 (36.2%) were 18–49 years old, 521 (12.4%) were 50–64 years of age, and 219 (5.2%) above the age of 65. Based on the data from the Vaccine Registry, 1433 (34.0%) vaccination statuses were identified. The data on vaccinations of those infected with a recombinant were as follows: there were 64 with one dose (4.5%), 358 with two doses (25.0%), 584 with three doses (40.8%), 179 with four doses (12.5%), and 71 with five doses (5.0%), and 177 were vaccinated after infection or without having been infected within 14 days of the earliest onset date (12.4%). It is important to note, however, that the timing and availability of vaccine doses changed throughout the pandemic (Supplemental Figure S1). Of those matched to the COVID-19 Hospitalization Registry and COVID-19 Case Registry, hospitalizations were reported for 51 (2.0%) cases infected with recombinant genomes, which included lineages XB, XBB, and XBB.1.5. Sixteen (0.6%) deaths occurred in cases with the recombinant lineages XAS, XB, XBB, XBB.1.5, and XE. 

Comparatively, in California, for all SARS-CoV-2 genomes (*n* = 801,534) sequenced prior to 1 January 2023, 24,483 (3.1%) hospitalizations and 4389 (0.5%) deaths occurred. Of these cases, 467,395 (58.3%) were unvaccinated or had an unknown vaccination status, and 19,099 (2.4%) had received four vaccine doses. It is important to note that these numbers only represent sequenced SARS-CoV-2 and not all cases.

### 3.3. Known Recombinant Lineages with Early Emergence in California

**Figure 3:**
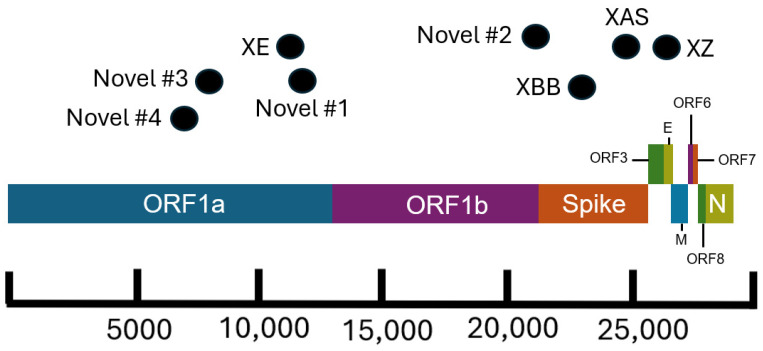
Schematic of the SARS-CoV-2 genome showing approximate breakpoint locations of novel and major recombinant lineages found in California. The horizontal location of the black dots represents the approximate region of the genome where recombination occurred for the corresponding recombinant lineages. Different colors represent different regions of the SARS-CoV-2 genome. The parental lineages of the recombinants are as follows: XE (BA.1 × BA.2), XZ (BA.2 × BA.1), XAS (BA.5 × BA.2), XBB (BA.2 × BA.2), Novel #1 (BA.5 × XBC.1), Novel #2 (BA.1 × BA.2), Novel #3 (BA.1 × BA.2), and Novel #4 (BA.1 × AY.44).

**Figure 4:**
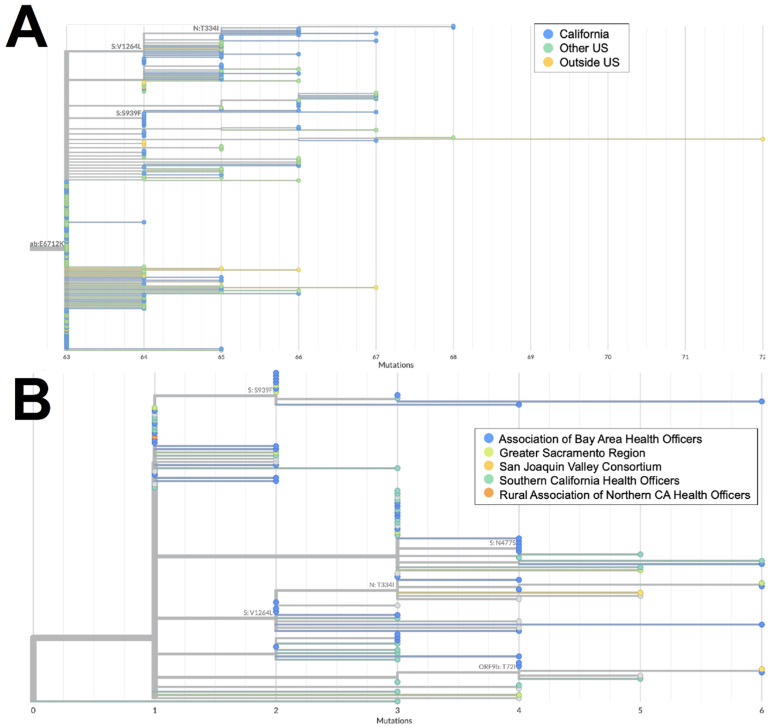
Phylogeny of XZ recombinant genomes. (**A**) Global UShER tree of XZ genomes from GISAID. Genomes are colored according to geographical location: in California (blue), outside of California but within the US (teal), or outside the US (orange). (**B**) Phylogenetic tree of XZ genomes within California from Terra and GISAID. Genomes are colored according to regional location: the Association of Bay Area Health Officers (blue), Greater Sacramento (green), San Joaquin Valley Consortium (light orange), South California Health Officers (teal), or Rural Association of Northern California Health Officers (dark orange). Genomes for which regional location information was not available are shown in gray.

Both XZ (*n* = 114) and XAS (*n* = 54) demonstrate early emergence in California. From March to July 2022, 122 genomes of the BA.2 × BA.1 recombinant XZ, with breakpoints identified in Figure 3, were identified in California. It is possible that this recombinant lineage originated in California, as many of the sequences with the earliest collection dates and that were present on the most ancestral branch were from California (Figure 4A), and sequences from California made up more than 50% of all XZ genomes from the United States. Within California, some of the most ancestral sequences came from multiple geographic regions (Figure 4B), indicating that this lineage was already widespread before it began to diversify. This particular lineage was also eventually identified in many other US states in March and April 2022; subsequently, it spread to Canada, Denmark, England, France, Germany, Mexico, and Japan from May through July 2022. 

**Figure 5:**
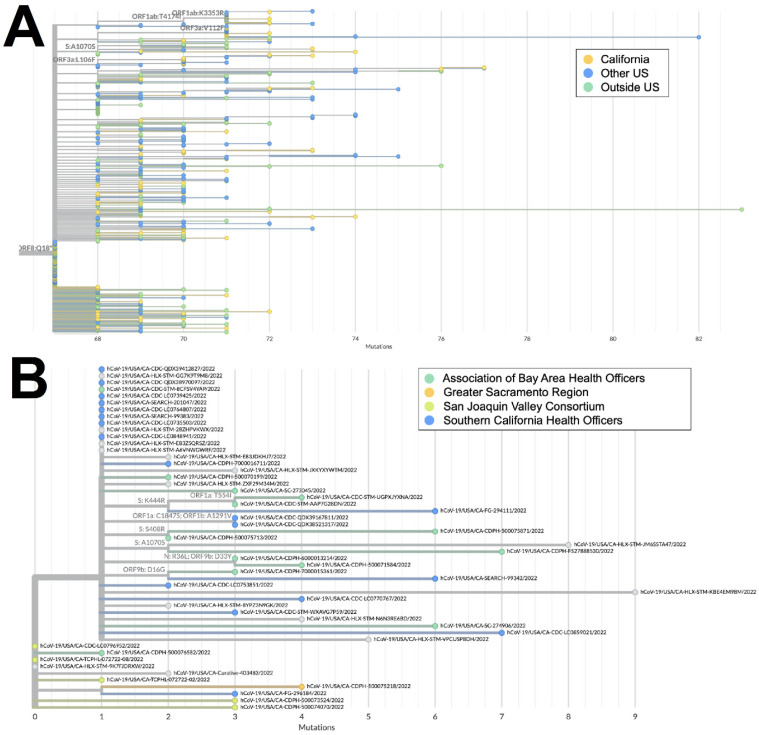
Phylogeny of XAS recombinant genomes. (**A**) Global UShER tree of XAS genomes from GISAID. Genomes are colored according to geographical location: California (orange), outside of California but within the US (blue), or outside the US (teal). (**B**) Phylogenetic tree of XAS genomes within California from GISAID. Genomes are colored according to regional location: the Association of Bay Area Health Officers (teal), Greater Sacramento (orange), San Joaquin Valley Consortium (green), or South California Health Officers (blue).

The XAS recombinant lineage had likely origins in Canada based on a large number of genomes from that country with early collection dates; it then spread to countries in North and South America as well as Europe. The sequences from California accounted for more than 40% of all the XAS genomes in the US and are disseminated throughout the global XAS tree (Figure 5A), indicating that this state played a key role in the propagation of this lineage within the US. Based on the phylogeny of sequences from California (Figure 5B), early emergence within the state occurred in the San Joaquin Valley Region, with a subsequent spread through Southern California and the Bay Area.

### 3.4. Novel Recombinant Lineages with Early Emergence in California

**Figure 6:**
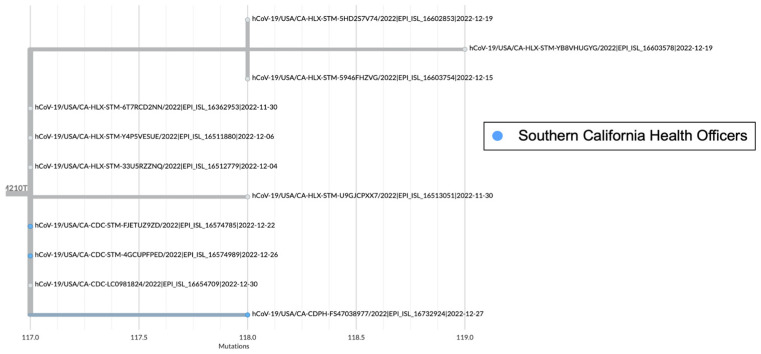
Global UShER tree of BA.5 × XBC.1 recombinant genomes from GISAID. Genomes from specimens originating in the Southern California Health Officers Region are shown in blue, and those for which regional location information was not available are shown in gray.

There are several recombinant lineages with early emergence in California that never grew beyond a small number of sequences and were either not proposed for or never received a Pango lineage designation. One of these novel recombinant lineages in California, hereafter designated as Novel #1, was first observed as a cluster of 11 BA.5 × XBC.1 sequences from four different sequencing laboratories (Figure 6). Novel #1 genomes had a recombinant site within ORF1a (Figure 3), and the XBC.1 portions of these genomes also contained an additional amino acid substitution in the N gene, N:M210T. The first two of these genomes originated from specimens collected on 30 November 2022, and genomes belonging to this novel recombinant lineage were still being identified through the end of 2022. This recombinant lineage was exclusive to sequences from California through the end of 2022. For the three sequences with granular location information available, all came from the Southern California Health Officers Region but from different counties within that region. 

Earlier in 2022, two novel and distinct BA.1 × BA.2 recombinant lineages were identified. The first cluster, designated as Novel #2, contained 11 BA.1 × BA.2 sequences from California collected on 15 February–18 March 2022 and 2 sequences from Nevada and Tennessee. The recombinant site for this cluster fell either in late ORF1b or early in the Spike protein based on the absence of the synonymous nucleotide mutation A20055G and the presence of S:T19I (Figure 3). A different BA.1 × BA.2 recombinant lineage, Novel #3, consisted of 18 genomes from California and one from Idaho, which were collected between February 18 and March 21, 2022. The recombinant site for this cluster fell within ORF1a based on the presence of ORF1a:L2084I and absence of ORF1a:A2710T (Figure 3).

Another recombinant cluster, Novel #4, contained a single Delta × Omicron recombinant genome from California (EPI_ISL_10378301), but eleven other genomes from at least four other states within the US were identified. The recombinant site was located within ORF1a, with the early part of the genome belonging to the Delta variant and potentially the AY.44 lineage within Delta based on the presence of synonymous nucleotide mutations early in the genome (Figure 3). The Omicron portion of the genome was determined to belong to the BA.1.1 lineage based on the presence of S:R346K amongst the many other Omicron-specific mutations. The California Novel #4 sequence originated from a sample collected on 13 February 2022 in Sacramento County that had an additional amino acid substitution, N:P364L, differentiating it from the 11 other genomes from Massachusetts, Utah, New Mexico, and Hawaii. 

### 3.5. Other Major Recombinant Lineages

The two largest recombinant lineages identified in California were XB (*n* = 886) and XBB (*n* = 2044), both of which likely had international origins. XB genomes were identified from March through September of 2021, and XB was the only recombinant lineage identified in California during the study period that did not have at least one Omicron parental lineage. 

The first XBB genome in California was collected on 27 July 2022, and this lineage remained in circulation through the end of 2022. This lineage diversified as it expanded globally, resulting in the XBB.1.5 sublineage, of which 419 genomes were identified in California during the study period.

## 4. Discussion

Geographic regions with higher population density and numbers of SARS-CoV-2 sequences in public repositories can provide insight into recombination events and the dissemination of recombinant lineages worldwide. On a global scale, recombinants were identified at a higher frequency later in the pandemic due to the increased co-circulation of divergent lineages [22]. Notably, in this study, although the percentage of cases sequenced decreased over time in California, there was an increase in recombinants detected (Figure 1). At least 30 different SARS-CoV-2 recombinant lineages were identified from California sequences collected through the end of 2022. California may be an ideal setting for the identification of emerging lineages and recombination events due to the population density of several of its metropolitan regions, the influx of international travelers via cross-border ports of entry and major international airports, and the co-circulation of multiple lineages. However, another possibility is that the high volume of sequencing performed in this state due to the California COVIDNet Initiative [13] increased the likelihood of detecting recombinant lineages in California compared to other geographic locations. 

Of the recombinants with matched case information, more than half originated in Southern California. This is not surprising considering that more than 22 million people (>50% of the population) reside in Southern California and that SARS-CoV-2 genomic surveillance of this region has been well represented. Interestingly, hospitalizations and deaths for individuals infected with recombinant genomes primarily occurred in the first 18 months of the pandemic, as seen with the lineage XB, which circulated from March through September of 2021. It is unclear if this observed difference is due to the lineage specifically or its emergence before COVID-19 vaccines were widely available. Had sample sizes been sufficient for statistical analyses, a comparison of COVID-19 severity between recombinant lineages and the parental lineages that gave rise to the recombinants may have helped to answer these questions. Regardless of the severity of COVID-19 outcomes, recombinants are important to surveil as their contribution to large mutational changes could lead to functional differences in transmissibility, immune escape, or pathogenesis. 

Identifying specific combinations of mutations that contribute to the success of recombinant lineages has major implications for forecasting which lineages might become dominant or be considered as targets for vaccine or drug development. The recombinant lineage XBB, initially detected in India and projected to become dominant worldwide at the end of 2022, contained many amino acid substitutions in the spike protein significantly associated with BA.2 breakthrough infection and enhanced ACE2 binding affinity [23]. However, contrary to predictions, XBB alone was not very advantageous on a global scale; it further diversified into XBB.1.5 with the additional S:G252V and S:F486P mutations that provided a substantial advantage against other lineages circulating at that time [24]. The recombinant lineage XAS had a partial BA.5 spike protein and perhaps, combined with the truncation of ORF8 from the BA.2 donor, a genomic feature hypothesized to be under positive selection, conferred an advantage over other circulating lineages for a brief period of time (https://virological.org/t/repeated-loss-of-orf8-expression-in-circulating-sars-cov-2-lineages/931, accessed 4 March 2024). Although the XZ recombinant contained the entire BA.2 spike protein, it also contained the positively selected M:D3G mutation from BA.1; the combination thereof may have conferred an advantage over the canonical BA.2 early in 2022 [25]. 

Tracking emerging lineages, including recombinants, that have not yet been assigned a Pango lineage presents a challenge for public health authorities trying to monitor the increase and decrease in the prevalence of certain lineages over time. There are also occasions where a lineage has been designated but cannot be assigned using the latest version of lineage-calling tools. Recently, there have been efforts to overcome this challenge, and web-based tools like UShER and Nextclade that are updated frequently have been transformative for SARS-CoV-2 genomic epidemiology [19,20]. However, many novel recombinant lineages that do not grow beyond a certain number of sequences remain undesignated. Retrospectively, it is understandable to attempt to avoid naming every cluster of sequences that diverge from an ancestor, but the challenge remains when these sequences appear in near real-time. Another challenge in characterizing recombinant lineages is the distorted tree topology that results from viewing recombinant and non-recombinant SARS-CoV-2 genomes on the same phylogenetic tree. This presents difficulties when trying to measure genomic divergence, as branch lengths are inflated relative to the true divergence from the parental lineages. Since contextual genomic data are crucial for the phylogenetic interpretation of emerging lineages, this does limit our ability to draw immediate conclusions about the origins of recombinants using traditional phylogenic methods. 

Analysis of wastewater has become increasingly critical for SARS-CoV-2 epidemiology following the shifting landscape of SARS-CoV-2 testing towards at-home rapid antigen tests instead of PCR tests. Metagenomic analyses of SARS-CoV-2 in wastewater relies on detecting a specific set of mutations in designated lineages known as barcodes. It is also more difficult to identify novel lineages including recombinants amidst the background signals of other SARS-CoV-2 lineages in wastewater samples, emphasizing a continued need for WGS data from clinical samples in combination with metagenomic data from wastewater. 

Some of the important limitations of this work apply to any study that aims to combine pathogen sequence data with epidemiologic data. Although epidemiologic information was included in this study, it was not available for every recombinant genome. When combined with the changes in reporting requirements as well as vaccine availability and recommendations throughout the pandemic, our ability to conduct statistical analyses and draw robust conclusions on associations between specific SARS-CoV-2 lineages and epidemiologic information was limited. While 59.9% of SARS-CoV-2 recombinant genomes matched to the CDPH databases, not all accompanying epidemiological information was available for each matched case due to data entry omissions and errors, staffing limitations, or changes in reporting requirements during the SARS-CoV-2 pandemic. The large proportion of sequences that did not match to CDPH databases may be due to differences between sample identifiers in the various databases. For example, virus names in GISAID that did not match identifiers in CDPH databases may have resulted in duplicate entries of samples or the inability to link to epidemiologic information for corresponding samples. Of the recombinant genomes in this study that did not match to the CDPH databases, 787 (46.6%) were identified from GISAID. Therefore, we were unable to link more than half of all the samples pulled from GISAID to the CDPH databases. Streamlining the naming schemes and reporting of testing results has proven to be difficult given the decentralized sequencing efforts in California. In the future, a standardized and automated system for linking sequence information from public repositories with epidemiologic records may improve public health surveillance efforts and allow for near real-time analyses of potential functional differences between SARS-CoV-2 lineages, including recombinants. 

There are additional limitations to consider when drawing conclusions based on the prevalence of SARS-CoV-2 recombinants in California, including (1) the lack of uniformity of sequencing capacity throughout the study period (Figure 1B), which may have affected the detection of recombinants; (2) the fact that sequence quality was not confirmed for samples from GISAID, which may have impacted the Pango lineage assignments; (3) the fact that retrospective submissions to GISAID after the study period with collection dates spanning from 2020 to 2022 may have resulted in their exclusion from this study; (4) the fact that California Case Registry hospitalization and death statuses do not differentiate between hospitalizations with and deaths due to COVID-19; and (5) the fact that we were unable to differentiate between unvaccinated cases and cases with an unknown vaccination status. 

Beyond the time period for this study, additional recombinants have emerged, and public health scientists have continued to assess the potential impact of new and divergent lineages. Recently, the world saw JN.1, a descendant of a second-generation BA.2 lineage, outcompete almost every other currently circulating lineage in a matter of months. However, previously circulating lineages have recombined with JN.1 to form new recombinants. Recently, XDP, a recombinant between JN.1 and FL.15 (XBB.1.9.1.15), has emerged and is now increasing in prevalence worldwide. While FL.15 is no longer in circulation and never grew beyond a few thousand sequences, its recombination with JN.1 in March 2024 gave rise to XDP, which was considered to have a growth advantage over JN.1. California COVIDNet continues to monitor XDP, as well as all SARS-CoV-2 recombinant lineages, within this state. 

This report highlights the successful detection and genomic characterization of recombinant SARS-CoV-2 lineages in California as well as remaining challenges for public health regarding how best to detect, monitor, and respond to novel lineages. These challenges will likely persist on a global scale, particularly in light of (1) the decreasing sequencing volumes following the expiration of public health emergencies worldwide, (2) the prevalence of at-home testing, (3) the increasing number of co-circulating lineages resulting in the potential for more recombinants (https://cov-lineages.org/lineage_list.html, accessed 4 March 2024), and (4) the continued convergent evolution of SARS-CoV-2 making it more difficult to distinguish between lineages. These challenges highlight the need to maintain SARS-CoV-2 genomic surveillance at a sufficient level to detect emerging lineages and recombinants, monitor changes in the virus, and inform public health responses and pharmaceutical interventions. 

## Figures and Tables

**Table 1:** Epidemiologic information associated with recombinant lineages in California.

Lineage	Dates Circulating in CA	Recomb ^^^ Lineages	*n*	Epi Data Available * (%)	Hospitalized (%)	Died (%)	Died w/2 or More Vaccine Doses	Age Range of Hospitalized or Died
All Recomb ^^^	--	--	4213	2523 (59.9%)	51 (2.0%)	16 (0.6%)	6	11–96
XB	Mar–September 2021	B.1.634 · B.1.631	886	497 (56.1%)	25 (5.0%)	7 (1.4%)	Unmatched	11–78
XE	Mar–July 2022	BA.1 · BA.2	161	106 (65.8%)	3 (2.8%)	3 (2.8%)	2	88–91
XZ	Mar–July 2022	BA.2 · BA.1	114	85 (74.6%)	2 (2.4%)	0	--	53–62
XAS	Jun–November 2022	BA.5 · BA.2	54	37 (68.5%)	1 (2.7%)	1 (2.7%)	1	75
XBB	August 2022-	BA.2 · BA.2	2044	1103 (54.0%)	9 (0.8%)	3 (0.2%)	2	69–96
XBB.1.5	November 2022-	BA.2 · BA.2	419	318 (75.9%)	6 (1.9%)	2 (0.6%)	1	29–84
Other Recomb ^^^	--	--	535	377 (70.5%)	5 (1.3%)	0	--	24–94

Recomb **^^^**: abbreviation of “Recombinant”. * Epi Data Available indicates the number of samples that matched to the COVID-19 Hospitalization Registry or COVID-19 Case Registry (epidemiologic data).

## Data Availability

Available sequencing data presented in the study are openly available in GISAID (https://gisaid.org/). GISAID Accessions are included in the Supplemental Table S1. Sequences not available in GISAID are available on request from the corresponding author due to the location information being considered identifiable or because GISAID had rejected the submitted genomes.

## Supplementary Materials

The following supporting information can be downloaded at https://www.mdpi.com/article/10.3390/v16081209/s1. Figure S1: Percentage of vaccinations over time; Table S1: List of available metadata for all samples.
