# Supplementary figures and images for: Emergence of Recombinant SARS-CoV-2 Variants in California from 2020 to 2022
Source: Viruses. 2024 Jul 27;16(8):1209. doi: 10.3390/v16081209 (PMC11359944; doi:10.3390/v16081209)

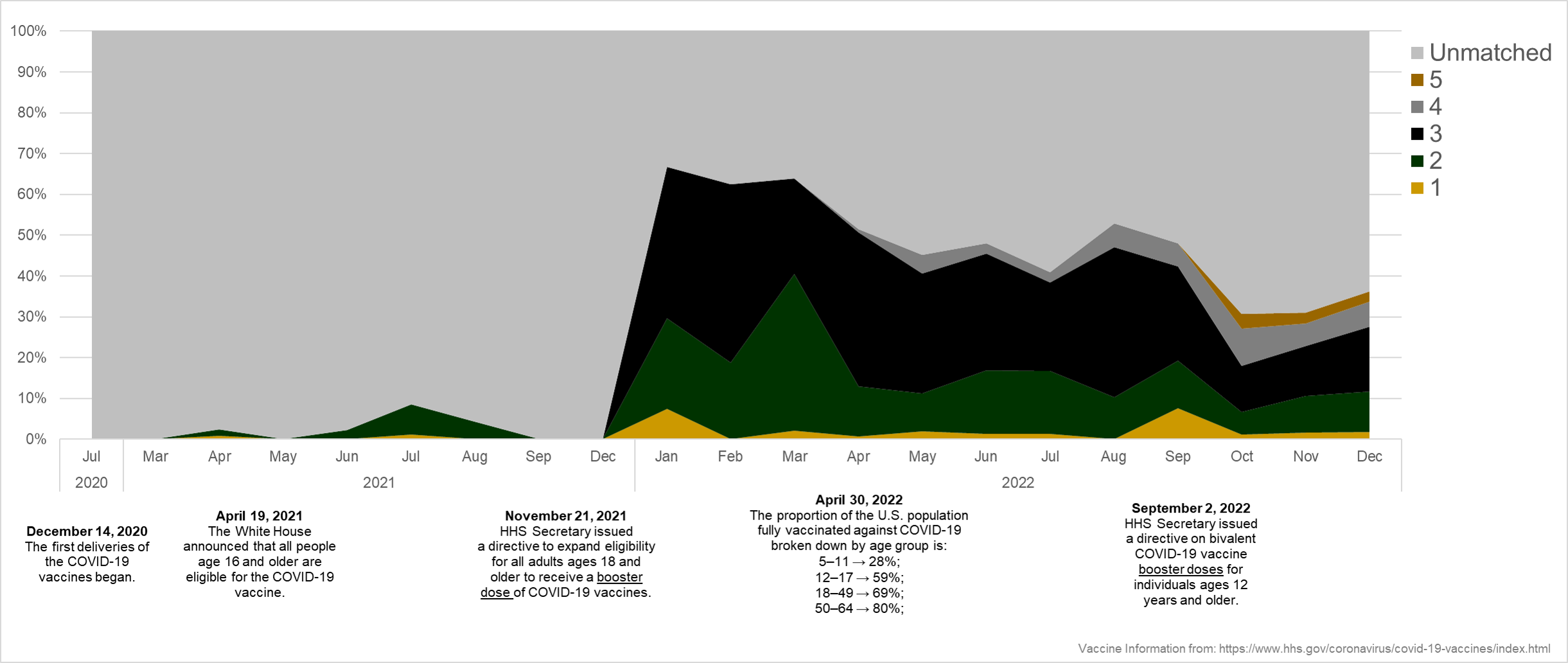

Supplement: Supplementary file 1 [file viruses-16-01209-s001.zip › Supplemental Figure S1.png]
